# Muscarynic metabotropic receptor M4 modulates the hippocampal CA1 LTP possibly through local GABAergic interneurons

**DOI:** 10.1186/1471-2202-15-S1-P63

**Published:** 2014-07-21

**Authors:** Querusche K Zanona, Flávia Z Boos, Ana Paula Crestani, Johanna M Duran, Maria E Calcagnotto, Jorge A Quillfeldt

**Affiliations:** 1 Neuroscience Graduate Program, Universidade Federal do Rio Grande do Sul, Porto Alegre, RS, 90040-060, Brazil; 2 Biochemistry Department, Universidade Federal do Rio Grande do Sul, Porto Alegre, RS, 90035-003, Brazil; 3 Biophysics Department, Universidade Federal do Rio Grande do Sul, Porto Alegre, RS, 90540-000, Brazil

## 

Muscarinic Cholinergic receptors of the subtype M4 are G protein coupled receptors that activate Gi reducing AMPc concentration on the cell and closing K^+^ channels, causing hyperpolarization of the cell membrane and reduction of neurotransmitter release [1]. Previous works from our laboratory using the very selective Muscarinic Toxin 3 (MT3) to block M4 receptors showed that infusion of this substance into the dorsal hippocampus impairs memory consolidation [2] and facilitates memory retrieval [3], suggesting an important role for M4 modulation of CA1 circuitry in memory processes. Also, local application of this toxin in hippocampal slices was able to impair long-term potentiation (LTP) [4]. One hypothesis raised to explain these findings suggests that plastic changes taking place in the CA1 circuitry involves differential control of local excitatory cells and inhibitory interneurons. Since changes in LTP can be elicited by different mechanisms, the objective of this work was to verify, through an analog of the pharmacological infusion used in the behavioral studies, the effect of MT3 on LTP and the activity of inhibitory interneurons as possible targets of this cholinergic action.

Thus, we recorded fEPSP from Schaffer collaterals at hippocampal CA1 area of rats anesthetized under urethane. After setting the position and the stimulation intensity to obtain 50% of maximum fEPSP, we recorded 10 min baseline followed by drug infusion (0.5μl, 20μl/h) through a delicate cannula glued to the recording electrode at the contralateral side of stimulae. We infused either MT3 (2.0μg), baclofen (0.1μg), MT3+baclofen (same doses), or their vehicles. After 15min, we applied a high frequency stimulation (HFS) protocol (10 trains at 0.5Hz of 20 pulses 100Hz) and recorded the fEPSP for at least 90min. None of the treatments affected the baseline excitability.

Our results indicate that: MT3 infusion 15min before HFS was able to block LTP, consistent with our previous finding with MT3 applied 2min before HFS [4], and baclofen, a GABA_B_ receptor agonist in a subeffective dose, was able to prevent this MT3 effect. Considering that hippocampal GABA_B_ receptors are mainly presynaptic and mostly located in GABAergic interneurons, and that its activation leads to cell hyperpolarization / reduction of GABA release, our results suggest that the M4 receptors relevant to those behavioral responses may be colocalized with GABA_B_ receptors in the CA1 interneurons. Further studies are necessary to verify this hypothesis.
